# Physical Activity, Nutrition, Cognition, Neurophysiology, and Short-Time Synaptic Plasticity in Healthy Older Adults: A Cross-Sectional Study

**DOI:** 10.3389/fnagi.2018.00242

**Published:** 2018-08-30

**Authors:** Alexandra Schättin, Federico Gennaro, Martin Egloff, Simon Vogt, Eling D. de Bruin

**Affiliations:** ^1^Department of Health Sciences and Technology, Institute of Human Movement Sciences and Sport, ETH Zürich, Zürich, Switzerland; ^2^Department of Neurobiology, Care Sciences and Society, Karolinska Institutet, Solna, Sweden

**Keywords:** older adults, synaptic plasticity, physical activity, nutrition, cognition, paired associative stimulation, transcranial magnetic stimulation, neurophysiology

## Abstract

The aging brain undergoes remodeling processes because of biological and environmental factors. To counteract brain aging, neuronal plasticity should be preserved. The aim of this study was to test if the capacity of generating short-time synaptic plasticity in older adults may be related to either physical activity, nutritional status, cognition, or neurophysiological activity. Thirty-six participants (mean age 73.3 ± 5.9 years) received transcranial magnetic stimulation in combination with peripheral nerve stimulation to experimentally induce short-time synaptic plasticity by paired associative stimulation (PAS). Adaptations in neuronal excitability were assessed by motor-evoked potential (MEP) in the right m. tibialis anterior before and after PAS. The Physical Activity Questionnaire 50+ and the StepWatch^TM^ captured physical activity levels. Nutritional status was assessed by the Mini Nutritional Assessment. Cognition was assessed by reaction time for a divided attention test and with the Montreal Cognitive Assessment. Neurophysiological activity was assessed by electroencephalography during the divided attention test. MEPs of the highest stimulation intensity resulted significantly different comparing before, 5 min, or 30 min after PAS (*p* < 0.05). Data-driven automatic hierarchical classification of the individual recruitment curve slopes over the three-time points indicated four different response types, however, response groups did not significantly differ based on physical activity, nutritional status, cognition, or neurophysiological activity. In a second-level analysis, participants having an increased slope showed a significant higher energy expenditure (*z* = -2.165, *p* = 0.030, *r* = 0.36) and revealed a significant higher power activity in the alpha frequency band (*z* = -2.008, *p* = 0.046, *r* = 0.37) at the prefrontal-located EEG electrodes, compared to the participants having a decreased slope. This study hints toward older adults differing in their neuronal excitability which is strongly associated to their short-time synaptic plasticity levels. Furthermore, a physically active lifestyle and higher EEG power in the alpha frequency band seem to be connected to the capacity of generating long-term potentiation-like synaptic plasticity in older adults. Future studies should consider more sensitive assessments and bigger sample sizes to get a broad scope of the older adults’ population.

## Introduction

Aging is a naturally occurring physiological process that develops without concurrent diseases (Mora, 2013). In humans, the central nervous system, in general, is affected by aging- and behavior-related alterations. This alteration process includes changes in brain neurochemistry as well as in neuroanatomical structures including gray and white matter (Park and Reuter-Lorenz, 2009; Seidler et al., 2010). Especially, gray and white matter in (pre)frontal and parietal cortices are susceptible to atrophy (Colcombe et al., 2003; Resnick et al., 2003; Seidler et al., 2010). White matter integrity decreases in an anterior-to-posterior gradient illustrated by diffusion tensor imaging (Head et al., 2004). Frontal and parietal cortices, including the motor and sensory cortical regions, are important for the realization of motor performance (Seidler et al., 2010; Blumen et al., 2018). Atrophy of the motor cortical regions may coincide with motor performance declines such as movement slowing, disorders in balance, and instable gait leading to fall events and fragility that, in turn, affect activity of daily living and maintenance of older adults’ independence (Seidler et al., 2010). One reason for successful (brain) aging might be to retain considerable neuronal plasticity, which leaves room for brain function adaptations triggered by lifestyle factors; e.g., physical activity and/or nutrition (Meeusen and De Meirleir, 1995; Carvalho et al., 2014). Thus, behavioral factors that might support successful (brain) aging and that might keep the aging brain adaptable to slow down the brain aging process need to be identified. Better understanding about brain plasticity might help older adults to counteract weakness, to remain independent and to get a balanced life (Taube et al., 2008).

Regarding brain aging, physical activity can strengthen neuronal structures, synaptic plasticity, and neuronal transmission (Cai et al., 2014). Physical activity seems to be essential in stimulating the transcription of neurotrophins, especially BDNF (Neeper et al., 1995; Flöel et al., 2010; Gomez-Pinilla et al., 2011; Sleiman et al., 2016). BDNF’s neurotrophic and neuroprotective properties include neuronal survival, neurite expression, and axonal as well as dendritic growth and remodeling (Lu and Figurov, 1997; Cowansage et al., 2010; Mora, 2013; Meeusen, 2014) and, therefore, BDNF modifies neuronal excitability (Figurov et al., 1996; Lu and Figurov, 1997). The maintenance of basal BDNF levels is needed for hippocampal neurogenesis (Yau et al., 2014). Older participants showed an increased hippocampal volume after chronic exercise (Erickson et al., 2009). Moreover, physically active older adults are better able to recruit additional brain resources to improve on various motor tasks than their sedentary counterparts (Seidler et al., 2010). Furthermore, older adults with a high aerobic fitness level have greater brain volumes in brain areas that are vulnerable to behavior-related effects (Colcombe et al., 2003). Thus, physical activity seems to be important for triggering molecular and cellular mechanisms in order to counteract age- and behavior-related decline in the brain (Cotman and Berchtold, 2002; Voss et al., 2013; Phillips et al., 2015).

Next to physical activity, also nutrition is believed to influence neurogenesis and synapse formation by altering neurotrophin levels, inflammation, and energy metabolism (Dauncey, 2009; Mora, 2013). Specific nutrients (e.g., uridine, docosahexaenoic acid, and choline) are needed for the formation of neuronal membranes and, thus, of synapses (Wurtman et al., 2009). Furthermore, several nutritional supplementations and/or dietary modifications may be able to prevent or postpone cognitive decline, e.g., omega-3 polyunsaturated fatty acids, amino acids, and polyphenols (Miquel et al., 2017; Phillips, 2017). Nevertheless, one should keep in mind that not a single nutrient seems to be essential, but a combination of several nutrients that interact to evoke beneficial and synergistic effects on the brain (von Arnim et al., 2010; Miquel et al., 2017). Mediterranean dietary including high consumption of fruits, vegetables, legumes, cereals, fish, and olive oil and low consumption of meat and dairy products could play a major role in cognitive health (Aridi et al., 2017). A recent study showed that a diet based on plant foods and fish was associated with better cognitive functions in older Japanese people (Okubo et al., 2017). Furthermore, the combination of a diet including health-supporting nutrients and physical activity could build a synergy effect on important molecular and cellular pathways for neurogenesis, cell survival, synaptic plasticity, and vascular function (van Praag, 2009). A recent review illustrated the importance of a combinatory approach including physical activity and nutrition in older adults (Schättin et al., 2016).

Cognitive status is a further factor that plays an important role in terms of aging. Older adults who engage in intellectually stimulating activities experience less hippocampal atrophy (Valenzuela et al., 2008). Enriched and/or stimulating environments upregulate BDNF and increase neurogenesis contributing to neuronal plasticity (van Praag et al., 1999, van Praag et al., 2000; Kempermann et al., 2002; Brown et al., 2003). Therefore, cognitive stimulation should be added to physical activity to promote the survival of newly born structures (Phillips, 2017). In older adults, cognitive dysfunction, especially disorders in executive functioning, plays a role in causing gait disturbance and fall events (Rapport et al., 1998; Scherder et al., 2011). Strengthened EFs, especially divided attention, might contribute to gait performance (Pichierri et al., 2012) and might reduce fall events as EFs performance predicted the risk for future falls (Mirelman et al., 2012). Moreover, performance in EFs was associated with larger prefrontal volume and greater PFC thickness in healthy adults (Yuan and Raz, 2014). During aging, the PFC is vulnerable to degenerative processes (Pfefferbaum et al., 2013; Fjell et al., 2014).

Regarding neurophysiology, previous research findings by means of EEG showed that aging is linked with a lowering of the individual peak of the EEG power within the alpha frequency band as well as a decrease of the latter and usually an increase of slow waves < ∼7 Hz (i.e., delta and theta frequency bands), so-called age-related “slowing” (Rossini et al., 2007; Ho et al., 2012). A reversal of this age-related process observed by means of EEG might be linked to ameliorated cognitive functioning, improved motor performance, and enhanced sensory processing (Rossini et al., 2007).

Indirect measures of strengthening the activity between neuronal populations may be used as a biomarker of synaptic plasticity (Voss et al., 2013). TMS can be used, in combination with PNS, to experimentally induce short-time synaptic plasticity by PAS (Cirillo et al., 2009) and can then be used to record adaptations by changes in neuronal excitability. With the use of TMS methodology, greater synaptic plasticity in the left abductor pollicis brevis muscle motor circuit could be related to more active young adults (age 18–38 years) (Cirillo et al., 2009). Information on the influence of physical activity, nutritional status, and cognition on synaptic plasticity in older adults focusing on the lower extremities is, however, lacking. This study, therefore, explores how either physical activity, nutritional status, cognition or neurophysiological activity by means of EEG might be related to synaptic functionality in elderly. A recent review summarized that several factors including increased physical activity, improved nutrition, and the performance of mental exercise can attenuate cognitive decline (Miquel et al., 2017). The aim of this study was to test whether the capacity of generating short-time synaptic plasticity in older adults may be related to either physical activity, nutritional status, cognition, or neurophysiological activity. We hypothesized that the capacity of generating short-time synaptic plasticity of the motor cortex is heterogeneous and this heterogeneity can be related to either physical activity, nutrition, cognition, or neurophysiological activity.

## Materials and Methods

### Study Design and Participants

This is a cross-sectional study that tested whether the capacity of generating short-time synaptic plasticity in older adults may be related to either physical activity, nutritional status, cognition, or neurophysiological activity by means of EEG. Potential participants were recruited through public advertisement on the homepage of the Senior University Zürich (Switzerland), in local senior residency dwellings (Zürich, Switzerland), and through study presentations in local elderly sports or leisure time groups (Zürich, Switzerland). The study was performed in the laboratory at the sport center ETH Hönggerberg (Zürich, Switzerland). Recruitment and measurements were executed within July 2015 and February 2016. The ethics committee of ETH Zürich, Switzerland (EK 2015-N-10) approved the study protocol. Before any measurements were performed, all eligible participants had to sign written informed consent according to the Declaration of Helsinki.

The potential participants had to be able to provide written informed consent and understand the study procedure and instructions. Moreover, the participants were screened for depression using the GDS (Yesavage et al., 1982; Yesavage and Sheikh, 1986) and the short EHI to determine the handedness of the participants (Oldfield, 1971; Veale, 2014). Furthermore, the participants completed a health questionnaire including questions about physical impairments, medical history, anthropometric data, and physical activity level. Participants fulfilling all of the following inclusion criteria were eligible for the study: (1) age ≥ 65 years, (2) live independently or in a residency dwelling, (3) non-smoker, (4) healthy (self-reported), (5) right-handed. Participants were excluded from the study, if they exhibit one of the following exclusion criteria: (1) mobility or cognitive impairments, (2) severe health problems (e.g., recent cardiac infarction, uncontrolled diabetes, or uncontrolled hypertension), (3) signs of an upcoming depression, (4) Alzheimer’s disease or another dementia, (5) rapidly progressive or terminal illness, (6) acute or chronic illness, (7) history of stroke or epilepsy, (8) history of seizure or individuals with a recent head injury, (9) medication that acts on neuronal level (psychotropic medications), (10) electronic or metallic head implants.

### Study Procedure

**FIGURE 1:**
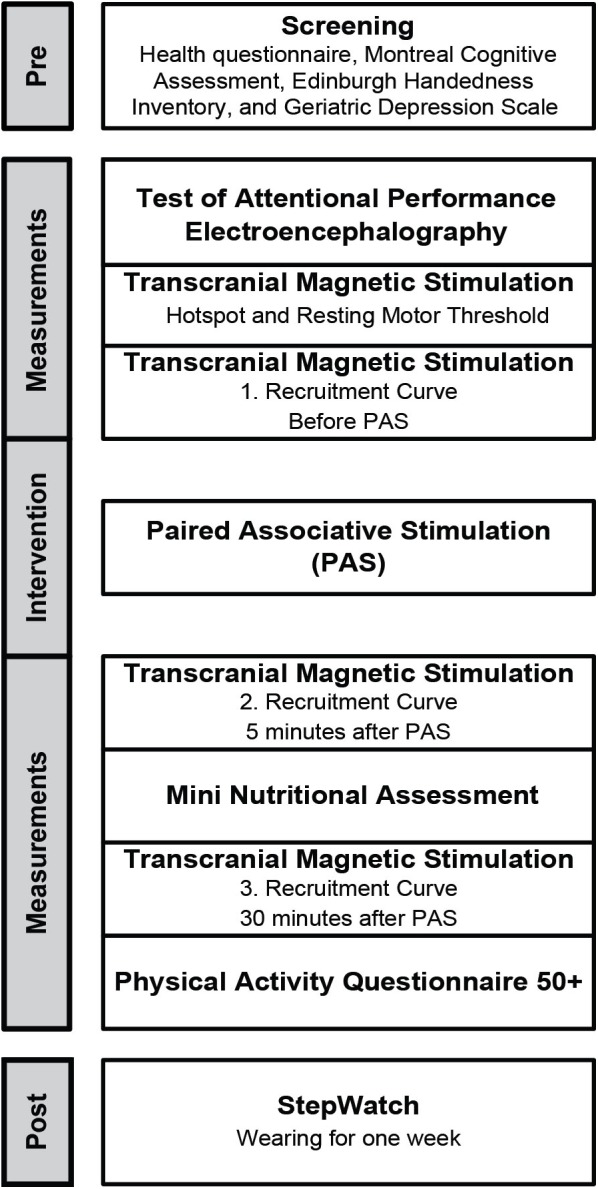
Study flow diagram.

**Figure 1** presents the study flow diagram that included screening, assessments to measure neuronal excitability, physical activity, nutritional status, cognition, neurophysiological activity by means of EEG, and PAS to elicit short-time synaptic plasticity.

### Measurements

#### Transcranial Magnetic Stimulation

On an adjustable chair, participants were seated comfortably with hip, knee, and ankle joint angles of 100, 120, and 90°, respectively. Only the dominant side, in this case the right side, was assessed because of the symmetrical nature of TMS-related measurements of the lower limb (Brouwer and Ashby, 1990). The right m. tibialis anterior was activated by stimulation over the motor cortex using a “figure-of-eight” coil by means of a TMS stimulator (Magstim Company, Ltd., Whitland, Dyfed, United Kingdom), using a previously established reliable TMS-related measurement protocol involving TA and healthy participants (Cacchio et al., 2009).

First, the participants got a bathing cap that fitted tight at the head. On the top of the cap, a grid was drawn using the vertex as initial position. The vertex was defined as half distance from nasion to inion and half distance from right to left pre-tragus. Detailed distance recordings were made from the nasion, inion, and bilateral pre-tragus to the vertex to maintain consistent coil positioning across sessions. The optimal activation of the TA seems to be obtained if the coil is placed parallel to and approximately 0.5–1.0 cm lateral to the midline and its mid-point is aligned anterior-posteriorly against the vertex (Cz) (Devanne et al., 1997). Second, the ideal stimulation point was assessed (hotspot). On the grid, the hotspot is where the threshold is lowest to evoke a MEP response on the TA (Rossini et al., 1994). Third, RMT was determined. RMT was the lowest intensity of magnetic stimulation required to evoke MEPs at TA with a peak-to-peak amplitude of minimum 50 μV, in at least 6 of 10 consecutive trials (Rossini et al., 1994).

Muscle activity of TA was recorded by Telemyo DTS (Noraxon, AZ, United States). For preparation, the skin of the shank was shaved (if needed) and prepared with an abrasive paste (OneStep AbrasivPlus, H+H Medizinprodukte, Münster, Germany). Afterward, two Ag/AgCl electrodes (Ambu Blue Sensor N, Cambridgeshire, United Kingdom) were placed on the muscle belly of the right TA (inter-electrodes distance of ∼2 cm). The EMG signals were high-pass filtered at 30 Hz. EMG data analysis was performed using MATLAB R2016a (The Mathworks, Natick, MA, United States). A time window encompassing the TMS impulse occurrence was used to identify the peak-to-peak amplitude of MEPs. For each stimulation intensity, the MEP was normalized using the baseline 140% RMT value of each individual participant.

##### Paired associative stimulation

At the level of synapse, no human neuroscience methods exist that can assess synaptic remodeling non-invasively. However, biomarkers of synaptic excitability/plasticity may represent indirect measures of acquisition and strengthening of activity between neuronal populations (Voss et al., 2013). PAS is a non-invasive approach to generate bidirectional changes in the excitability of the cortical projections to the muscles. PAS is a protocol involving TMS in combination with PNS to experimentally induce short-term synaptic plasticity (Cirillo et al., 2009). The PAS-based plastic changes of the cortical synaptic activity shows properties that seem to be similar to LTP (Bi and Poo, 1998) as rapid onset, associativity, duration, specificity, and NMDA-receptor dependence (Cooke and Bliss, 2006; Ziemann et al., 2008).

Within the PAS protocol of this study, PNS consisted of a percutaneous single electrical stimulation of the right common peroneal nerve delivered at 300% of the perceptual threshold followed by a single TMS pulse (120% RMT) 55 ms later over the motor cortex. The ISI was determined based on a previous study where an ISI of 55 ms was found to be optimal for inducing the greatest increase in the TA MEP amplitude for PAS during rest (Mrachacz-Kersting et al., 2007). A total of 90 stimuli were applied at a rate of 0.05 Hz lasting about 30 min. A constant current stimulator (DS7A stimulator, Digitimer, Ltd., Welwyn Garden City, United Kingdom) was used to apply electrical stimuli as squared wave pulses with a pulse width of 200 μs. Bipolar surface electrodes, separated by 30 mm, were placed on the right common peroneal nerve. As PAS induced plasticity on hand muscles seems to be connected to attention-dependent activation of the ascending cholinergic system (Stefan et al., 2004; Ziemann et al., 2008), our participants had to count the sensory stimuli during the PAS protocol to minimize the influence of this factor.

##### Recruitment curve

Recruitment curve was performed before PAS, 5 min after PAS, and 30 min after PAS. RC was obtained from 90% RMT to 140% RMT by increasing intensities in 10% steps. The intensities were applied in a random order (Möller et al., 2009). Each stimulation intensity contained 10 stimuli including a stimulus interval of 7 s with a 20% variance to avoid familiarization. The slope of the RC was calculated as the slope of the linear regression line through a supplied set of x-values (normalized MEP values) and y-values (stimulation intensities). An *a priori* data-driven strategy was used in order to subgroup the participants based on the PAS-response. That is, subgrouping was performed by an automatic hierarchical two-steps classification, taking into account: (1) the increase or decrease of the RC slope 5 min after PAS compared to baseline; (2) the increase or decrease of the RC slope 30 min after PAS compared to 5 min after PAS.

#### Physical Activity

##### Physical Activity Questionnaire 50+

The PAQ 50+ retrospectively quantifies the physical activity of older adults performed per week during the last month. The German PAQ 50+ shows good reliability for the total time of physical activity (*r* = 0.60) and for the total energy expenditure (*r* = 0.52) (Huy and Schneider, 2008). The questionnaire is based on the Yale Physical Activity Surveys and the Physical Activity Scale for the Elderly, which both show good test validity (Dipietro et al., 1993; Washburn et al., 1993; Montoye et al., 1996; Harada et al., 2001). The 37 questions cover activities including the household, gardening, leisure activities, sports, activity during work, and anthropometrical information (age, sex, height, and weight). Each activity is assessed by determining a certain number of MET-values. Through an equation involving the specific activity/MET-value, the duration of activity performance, and the weight of the person, the energy expenditure (kcal per week) was calculated. The sum of all activities equals the total score of the PAQ 50+ in kilocalories per week.

##### StepWatch*^TM^*

The StepWatch (modus health IIc, Washington, DC, United States) is an accelerometer attached to the ankle measuring the daily physical activity range. The StepWatch is a valid device to assess physical activity in community-dwelling older adults (Bergman et al., 2008). At slow walking speed, the StepWatch was more accurate than other activity monitors (Storti et al., 2008). The accuracy of the StepWatch is high with 96.06% for indoor use and 99.58% for outdoor use, respectively. Reliability was reported with an intra-class correlation coefficient of 0.91 (Mudge et al., 2010). Each participant wore the accelerometer for 1 week after the measurement procedure. StepWatch software was used to discern the average amount of steps per day for each participant.

#### Nutritional Status

##### Mini Nutritional Assessment

The MNA is a questionnaire evaluating the nutritional status (Guigoz et al., 1996). In total, 24 to 30 points are equivalent to a normal nutritional status, 17 to 23.5 points to being at risk of malnutrition, and less than 17 points means to be malnourished. The MNA is well-validated and correlates highly with objective indicators of nutritional status and clinical assessment (Guigoz et al., 2002). The scale has been used to assess the nutritional risk of older adults (Guigoz et al., 2002).

#### Cognition

##### Test of Attentional Performance

The D-TAP 2.3 VL (PSYTEST, Psychologische Testsysteme, Herzogenrath) is a valid test battery to assess deficits in attention and the subtests measure independent attentional functions (Zimmermann and Fimm, 2002). In this study, the focus was on the divided attention subtest lasting about 3 min. The test consists of concurrent appearance of visual and acoustic signals resulting in reaction time for key stimuli.

##### Montreal Cognitive Assessment

The MoCa assesses multiple cognitive domains including memory, language, EFs, visuospatial skills, calculation, abstraction, attention, concentration, and orientation (Nasreddine et al., 2005; Julayanont et al., 2013). The participants can get a maximum of 30 points while 22 points are a cutoff for mild cognitive impairment. The MoCa is a reliable (*r* = 0.75) and valid quantitative estimate of overall cognitive abilities (Koski et al., 2009).

#### Neurophysiological Functioning

##### Electroencephalography

Electroencephalography measurements were performed during the divided attention subtest of the TAP. After proper skin cleansing and preparation by applying an abrasive skin paste (NuPrep, Weaver and Company, Aurora, CO, United States), EEG activity was recorded using two Ag/AgCl sensors (Ambu White Sensors, Ambu A/S, Denmark) placed over the left and right PFC, respectively Fp1 and Fp2, according to the international 10/20 electrodes placement system and sampled at 128 Hz (Alpha-Active, Ltd., Devon, United Kingdom). The reference and ground were settled up by placing a passive CMS electrode at Fpz and two active DRL over the left and right mastoid, respectively. EEG data analysis was performed using custom scripts written in MATLAB R2017b (The Mathworks, Natick, MA, United States) and jointly using functions from EEGLAB 14.1.0b (Delorme and Makeig, 2004), ERPLAB (Lopez-Calderon and Luck, 2014) and Fieldtrip (Oostenveld et al., 2011) open source toolboxes. First, raw EEG data were high-pass filtered [zero-phase Hamming windowed sinc FIR, order: 425, cut-off frequency (-6 dB): 0.5 Hz, passband edge: 1 Hz, transition bandwidth: 1 Hz] and then low-pass filtered [zero-phase Hamming windowed sinc FIR, order: 59, cut-off frequency (-6 dB): 33.75 Hz, passband edge: 30 Hz, transition bandwidth: 7.5 Hz]. Then, EEG data were epoched in segments of 1 s duration time locked from 0.25 s before to 0.75 s after either auditory or visual stimulus onset. The total number of EEG data segments for each participant was 29 (15 relative to auditory stimuli and 14 relative to visual stimuli). Each entire EEG epoch, separately for auditory and visual stimuli segments and in each participant, was automatically checked for the presence of artifacts (e.g., eye blinks, ocular movements) by means of an absolute peak-to-peak voltage threshold criterion detection of ± 100 μV within a 200 ms width sliding window (100 ms steps). Subsequently, the accepted data segments were further checked and rejected when they exceeded both locally and globally the average probability of ±5 standard deviation (SD). For each participant, if a data segment relative to either Fp1 or Fp2 electrode was marked for rejection, the EEG epochs relative to both channels were rejected. Additionally, a rule of 50% maximum of rejected epochs was applied for each subject, removing the entire participant from further analysis in case this threshold was reached. Six participants were removed using these criteria (in both auditory and visual EEG epochs) and, on average, ∼13 (∼84% of each participant; SD: ∼15%; range: ∼53–100%) and ∼12 (∼84% of each participant; SD: ∼13%; range: ∼50–100%) total epochs remained for further analysis of the auditory and visual EEG data segments, respectively. For spectral EEG analysis, a multitaper Fast Fourier Transform was then employed in order to obtain the theta (4–8 Hz) and alpha (9–13 Hz) power spectrum by using a single Hanning taper with a frequency resolution of 1 Hz. The achieved individual theta and alpha power spectrum was log-transformed and averaged.

### Statistical Analysis

Due to non-normality of data, non-parametric tests were used. Outliers were replaced with a value that was the mean and two times standard deviation. Friedman test was used to compare the peak-to-peak amplitudes of the same stimulation intensities and the slopes of the RCs over the three measurement time points. Dunn–Bonferroni test was used for *post hoc* comparisons. Comparisons for physical activity, nutritional status, cognition, or neurophysiological activity between different subgroups obtained from the abovementioned data-driven automatic hierarchical classification were performed using Kruskal–Wallis test or Mann–Whitney *U*-test. Effect sizes were calculated and expressed as *r* = Z/√N. An effect size of *r* = 0.1 is considered a “small” effect, around 0.3 a “medium” effect, and 0.5 and above a “large” effect. A probability level of α = 0.05 was considered to be statistically significant. All analyses were conducted using SPSS Version 24.0 for Windows (SPSS, Inc., Chicago, IL, United States).

## Results

The recruitment phase resulted in 75 potentially interested elderly who received further study information. 36 older adults were included while 39 either declined participation or exhibited exclusion criteria. **Table 1** summarizes characteristics of the included participants.

**Table 1:** Participants’ characteristics and test results.

		*N* = 36	Female *N* = 20	Male *N* = 16
Age	years	73 (67; 78.3)	72 (67; 77.25)	74.5 (68; 79.75)
Height	meter	1.66 (1.59; 1.74)	1.61 (1.56; 1.65)	1.75 (1.71; 1.82)
Weight	kg	69 (60; 79.25)	66 (54.25; 73.50)	72 (65.75; 86.25)
BMI	kg/m^2^	24 (21.4; 28.2)	25.5 (20.9; 29.1)	23.6 (21.8; 26.5)
Education		2 (2; 4)	2 (2; 3)	2.5 (2; 4)
GDS	points	0 (0; 1)	0.5 (0; 1)	0 (0; 1)
RMT	%	45 (42; 50)	46 (43; 50)	45 (37.25; 49.5)

MoCA	points	25 (23; 27)	26 (23; 27.75)	24 (23; 26.75)
TAP	reaction time [ms]	760.3 (692.1; 816.6)	763.5 (689.4; 801.3)	760 (692.5; 833.8)
MNA	points	27 (26; 28.5)	26.5 (26; 28.5)	27.3 (24.4; 28.4)
PAQ 50+	kcal/week	6095 (4589; 9880.75)	6148 (4645.25; 9665.5)	5792 (4381.25; 11057.25)
StepWatch	steps/day	7270 (5522; 8476)^1^	7509 (5547; 8801.5)	6919 (5417; 7539)^2^

EEG	log-transformed power	*N* = 29	*N* = 17	*N* = 12
**Auditory stimuli**				
Theta Fp1		0.56 (0.49; 0.69)	0.60 (0.51; 0.74)	0.51 (0.42; 0.71)
Theta Fp2		0.45 (0.32; 0.57)	0.53 (0.37; 0.63)	0.38 (0.17; 0.50)
Alpha Fp1		0.46 (0.29; 0.61)	0.53 (0.35; 0.69)	0.34 (0.19; 0.56)
Alpha Fp2		0.35 (0.22; 0.56)	0.36 (0.22; 0.59)	0.31 (0.10; 0.59)
**Visual stimuli**				
Theta Fp1		0.56 (0.42; 0.67)	0.62 (0.46; 0.70)	0.49 (0.36; 0.67)
Theta Fp2		0.44 (0.32; 0.60)	0.46 (0.41; 0.65)	0.37 (0.25; 0.56)
Alpha Fp1		0.48 (0.28; 0.63)	0.49 (0.37; 0.64)	0.34 (0.19; 0.64)
Alpha Fp2		0.28 (0.20; 0.51)	0.37 (0.23; 0.58)	0.22 (0.14; 0.49)


*Data are median values (interquartile range) as indicated. Education indicates highest achieved graduation: 1 = primary school, 2 = apprenticeship/vocational school, 3 = high school and 4 = university. ^1^*N* = 35, ^2^*N* = 15. BMI, Body Mass Index; EEG, electroencephalography; GDS, Geriatric Depression Scale; MNA, Mini Nutritional Assessment; MoCA, Montreal Cognitive Assessment; PAQ, Physical Activity Questionnaire; RMT, resting motor threshold; TAP, Test of Attentional Performance.*

### Comparison of MEP Peak-to-Peak Amplitudes and RC Slopes

**FIGURE 2:**
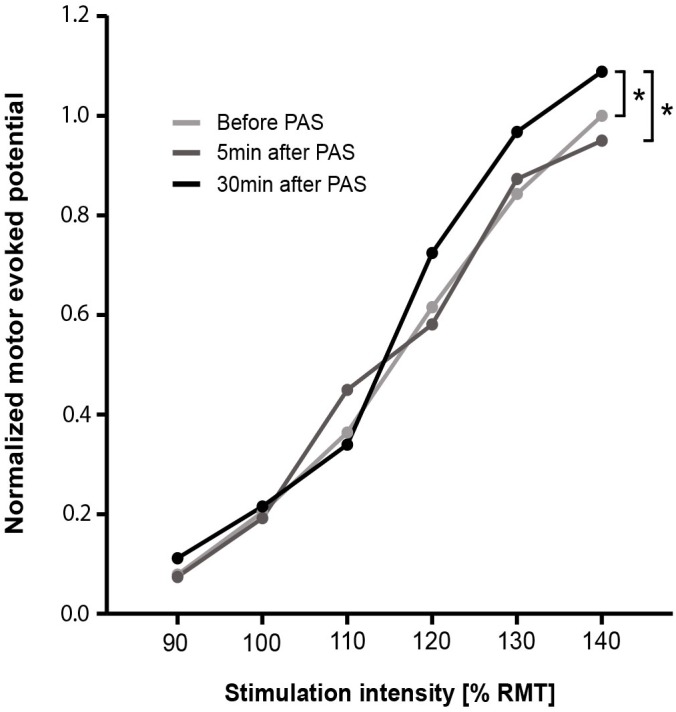
Recruitment curve of normalized MEP peak-to-peak amplitudes for before, 5 min after, and 30 min after PAS. Data points are median values. For each stimulation intensity, the comparison of before, 5 min after, and 30 min after PAS was performed using Friedman test. Dunn–Bonferroni *post hoc* test was performed when a significant comparison was analyzed. ^∗^*p* < 0.05. PAS, paired associative stimulation; RMT, resting motor threshold.

Three TMS stimulation intensities spread over two participants were not well-recorded and, thus, were not considered for analysis. The RC of normalized MEP peak-to-peak amplitudes over all three-time points is presented in **Figure 2**. Comparing MEP peak-to-peak amplitudes over all three-time points, significant differences were found for 130% RMT [χ^2^(2) = 6.000, *p* = 0.050, *n* = 36] and 140% RMT [χ^2^(2) = 13.556, *p* = 0.001, *n* = 36] (**Supplementary Table 1**). *Post hoc* test showed no significant differences for 130% RMT. *Post hoc* test for 140% RMT showed significant higher MEP peak-to-peak amplitudes for PAS_30__minafter_ compared to PAS_before_ (*z* = -3.064, p_adapted_ = 0.007, *r* = 0.39) and compared to PAS_5__minafter_ (*z* = -3.300, p_dapted_ = 0.003, *r* = 0.36).

**FIGURE 3:**
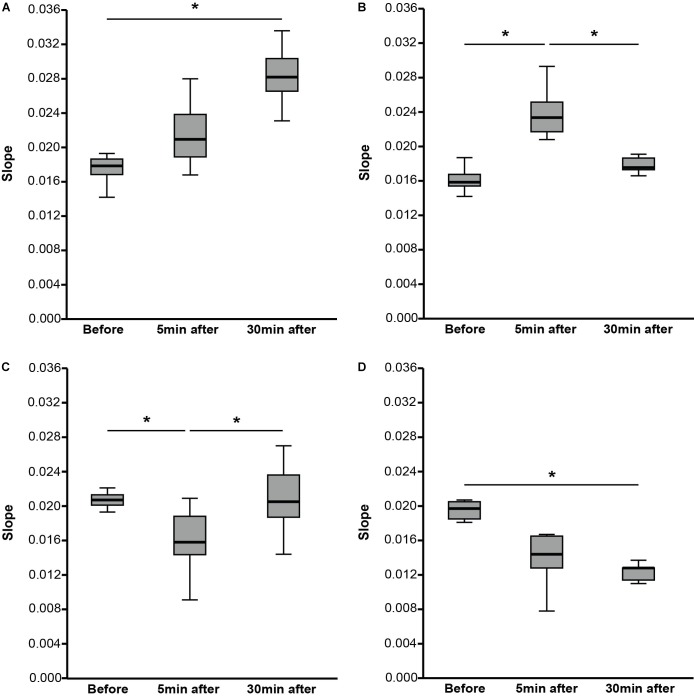
Slope of recruitment curves (RCs) for before, 5 min after, and 30 min after PAS divided into four different response groups. **(A)** The participants (*N* = 8) showed an increase of the slope from before to 5 min after PAS and to 30 min after PAS. **(B)** The participants (*N* = 8) showed an increase of the slope from before to 5 min after PAS followed by a decrease of the slope from 5 to 30 min after PAS. **(C)** The participants (*N* = 15) showed a decrease of the slope from before to 5 min after PAS followed by an increase of the slope from 5 to 30 min after PAS. **(D)** The participants (*N* = 5) showed a decrease of the slope from before to 5 min after PAS and to 30 min after PAS. Data are median values (interquartile range) as indicated. The slope of the RC was calculated as the slope of the linear regression line through a supplied set of x-values (normalized motor-evoked potential values) and y-values (stimulation intensities). For each group, the slope comparisons of before, 5 min after, and 30 min after PAS were performed using Friedman test. Dunn–Bonferroni *post hoc* test was performed when a significant comparison was analyzed. ^∗^*p* < 0.05.

Comparing the slope of the RCs over all three-time points, no significant difference was found [χ^2^(2) = 4.408, *p* = 0.110, *n* = 36] (**Table 2**). The data-driven automatic hierarchical classification of the individual RC slopes over the three-time points indicated four different response types to the PAS protocol (**Figure 3**). The results of the RC slope within group comparisons are presented in **Table 2**.

**Table 2:** Comparison of recruitment curve slopes for before, 5 min after, and 30 min after paired associative stimulation over all the participants and for the four different response groups.

	Before	5 min after	30 min after	χ^2^	df	*p*
**Overall**	0.019 (0.016; 0.021)	0.019 (0.015; 0.022)	0.020 (0.017; 0.026)	4.408	2	0.110

**Group A**	0.018 (0.017; 0.019)	0.021 (0.019; 0.024)	0.028 (0.027; 0.031)	15.548	2	<0.001^∗^
***Post hoc***	***z***	**p_adapted_**	***r***			
Before – 5 min after	-1.750	0.240	0.44			
5 min after – 30 min after	-2.125	0.101	0.53			
Before – 30 min after	-3.875	<0.001^∗^	0.97			

**Group B**	0.016 (0.015; 0.017)	0.023 (0.021; 0.026)	0.018 (0.017; 0.019)	13.000	2	0.002^∗^
***Post hoc***	***z***	**p_adapted_**	***r***			
Before – 5 min after	-3.500	0.001^∗^	0.88			
5 min after – 30 min after	2.500	0.037^∗^	0.63			
Before – 30 min after	-1.000	0.317	0.25			

**Group C**	0.021 (0.020; 0.022)	0.016 (0.014; 0.019)	0.021 (0.018; 0.024)	22.533	2	<0.001^∗^
***Post hoc***	**z**	**p_adapted_**	***r***			
Before – 5 min after	4.199	<0.001^∗^	0.77			
5 min after – 30 min after	-4.017	<0.001^∗^	0.73			
Before – 30 min after	0.183	0.999	0.03			

**Group D**	0.020 (0.018; 0.021)	0.014 (0.010; 0.017)	0.013 (0.011; 0.013)	8.316	2	0.016^∗^
***Post hoc***	***z***	**p_adapted_**	***r***			
Before – 5 min after	2.055	0.119	0.65			
5 min after – 30 min after	0.632	0.999	0.20			
Before – 30 min after	2.688	0.022^∗^	0.85			


*Overall: N = 36, group A: N = 8, group B: N = 8, group C: N = 15, group D: N = 5. Data are median values (interquartile range) as indicated. The slope of the recruitment curve was calculated as the slope of the linear regression line through a supplied set of x-values (normalized MEP values) and y-values (stimulation intensities). For each group, the slope comparisons of before, 5 min after, and 30 min after PAS were performed using Friedman test. Dunn–Bonferroni post hoc test was performed when a significant comparison was analyzed. ^∗^p < 0.05. For effect size, r = 0.1 is considered a “small” effect, around 0.3 a “medium” effect, and 0.5 and above a “large” effect.*

### Physical Activity, Nutritional Status, Cognition, and Neurophysiological Functioning

Due to technical problems, StepWatch^TM^ data of one participant and EEG data of one participant were not recorded. The results of the assessments for each response group are listed in **Tables 3, 4**. The comparison of each assessment between the four response groups revealed no significant differences (**Tables 3, 4**). Considering an additional sub-analysis, the comparison of the two groups (Groups A and B) classified by their increased RC slope with the two groups (Groups C and D) classified by their decreased RC slope, showed a significant difference for PAQ 50+ (*z* = -2.165, *p* = 0.030, *r* = 0.36). The groups having an increased slope [median (IQR): 8834.5 (5107; 11661) kcal/week] showed a higher energy expenditure than the groups having a decreased RC slope [median (IQR): 5345.5 (4365.5; 7207) kcal/week]. Furthermore, the log-transformed alpha power at Fp1 during auditory stimuli in the divided attention test led to a significant difference between the same abovementioned groups (*z* = -2.008, *p* = 0.046, *r* = 0.37). The groups having an increased RC slope showed a higher power in the alpha frequency band [median (IQR): 0.53 (0.36; 0.70) log-transformed power] compared to the groups having a decreased RC slope [median (IQR): 0.33 (0.25; 0.56) log-transformed power].

**Table 3:** Comparison of physical activity, nutritional status, and cognition between the different response groups.

	MNA [points]	MoCA [points]	TAP [reaction time, ms]	PAQ50+ [kcal/week]	StepWatch [Ø steps/day]
**Response groups**					
**A**	27 (23.8;27.9)	26.5 (23.25; 28.5)	749 (689.5; 807.1)	5679.5 (4591.8; 11549.5)	6503 (4986.5; 7525.5)
**B**	26.75 (24.6; 28.4)	25.5 (20.75; 27.75)	744 (660.6; 978.3)	10188 (6457; 12600.5)	7610 (7030; 9330)^1^
**C**	28 (26; 29)	25 (23; 26)	785 (692.5; 822.5)	6325 (4400; 8322)	7231 (4879; 8204)
**D**	26.5 (24.8; 27.3)	23 (22.5; 26.5)	745.5 (663.3; 781.7)	4631 (3162.5; 5748.45)	6915 (5310.5; 9516)

**Comparison between the four response groups**					
**χ^2^(3)**	4.294	1.671	2.186	7.536	3.320
***p***	0.231	0.643	0.535	0.057	0.345

**Comparison criteria slope** (Groups A and B *N* = 16 vs. Groups C and D *N* = 20)					
***z***	–1.056	–1.026	–0.621	–2.165	–0.367^2^
***p***	0.305	0.320	0.539	0.030^∗^	0.730^2^
***r***	0.18	0.17	0.10	0.36	0.06^2^


*Overall: N = 36, group A: *N* = 8, group B: *N* = 8, group C: *N* = 15, group D: *N* = 5. Data are median values (interquartile range) as indicated. For each assessment, the between group comparison (all groups) was performed using Kruskal–Wallis test. For each assessment, the comparison between two groups was performed using Mann–Whitney *U*-test. ^∗^*p* < 0.05. For effect size, *r* = 0.1 is considered a “small” effect, around 0.3 a “medium” effect, and 0.5 and above a “large” effect. ^1^*N* = 7, ^2^Groups A and B *N* = 15. MNA, Mini Nutritional Assessment; MoCA, Montreal Cognitive Assessment; TAP, Test of Attentional Performance; PAQ, Physical Activity Questionnaire.*

**Table 4:** Comparison of prefrontal cortex log-transformed power in theta and alpha frequency bands during the divided attention test between the different response groups.

Visual				
	**Theta**		**Alpha**	
	**Fp1**	**Fp2**	**Fp1**	**Fp2**

**Response group**				
A	0.50 (0.36; 0.57)	0.41 (0.36; 0.46)	0.64 (0.23; 0.80)	0.42 (0.14; 0.47)
B	0.54 (0.47; 0.72)	0.46 (0.32; 0.61)	0.44 (0.31; 0.63)	0.32 (0.21; 0.54)
C	0.59 (0.40; 0.75)	0.45 (0.28; 0.62)	0.48 (0.19; 0.55)	0.23 (0.15; 0.55)
D	0.63 (0.42; 0.70)	0.55 (0.31; 0.66)	0.47 (0.33; 0.64)	0.47 (0.30; 0.64)

**Comparison between the four response groups**				
**χ^2^(3)**	1.692	0.633	1.038	2.330
***p***	0.639	0.889	0.792	0.507

**Comparison criteria slope** (Groups A and B *N* = 13 vs. Groups C and D *N* = 16)				
*z*	–0.965	–0.526	–0.526	–0.263
*p*	0.351	0.619	0.619	0.812
*r*	0.18	0.10	0.10	0.05

**Auditory**				

	**Theta**		**Alpha**	
	**Fp1**	**Fp2**	**Fp1**	**Fp2**

**Response groups**				
A	0.60 (0.49; 0.72)	0.45 (0.35; 0.53)	0.55 (0.37; 0.61)	0.41 (0.21; 0.61)
B^1^	0.62 (0.52; 0.80)	0.53 (0.44; 0.60)	0.50 (0.36; 0.76)	0.43 (0.34; 0.62)
C^2^	0.56 (0.40; 0.69)	0.39 (0.31; 0.67)	0.33 (0.25; 0.66)	0.27 (0.14; 0.35)
D	0.54 (0.74; 0.43)	0.41 (0.25; 0.70)	0.41 (0.20; 0.70)	0.36 (0.04; 0.66)
**Comparison between the four response groups**				
χ^2^(3)	1.655	2.226	4.101	3.921
*p*	0.647	0.527	0.251	0.270

**Comparison criteria slope** (Groups A and B *N* = 14 vs. Groups C and D *N* = 15)				
***z***	–0.829	–0.698	–2.008	–1.877
***p***	0.425	0.505	0.046^∗^	0.063
***r***	0.15	0.13	0.37	0.35


*Overall: N = 29, group A: *N* = 7, group B: *N* = 6, group C: *N* = 12, group D: *N* = 4. Normalized data are median values (interquartile range) as indicated. For each assessment, the between group comparison (all groups) was performed using Kruskal–Wallis test. For each assessment, the comparison between two groups was performed using Mann–Whitney *U*-test. ^∗^*p* < 0.05. For effect size, *r* = 0.1 is considered a “small” effect, around 0.3 a “medium” effect, and 0.5 and above a “large” effect. ^1^*N* = 7, ^2^*N* = 11.*

## Discussion

The aim of this study was to test whether the capacity of generating short-time synaptic plasticity in older adults may be related to either physical activity, nutritional status, cognition, or neurophysiological activity. The results showed a significant increase of the TA MEP peak-to-peak amplitudes for the highest stimulation intensity comparing 30 min after PAS to before PAS as well as to 5 min after PAS. The data-driven automatic hierarchical classification of the individual slopes over the three-time points led to the identification of four different response types to the PAS protocol. The comparisons of the assessments including physical activity, nutritional status, cognition, and neurophysiological activity between the response groups, however, revealed no significant differences between these groups. Nevertheless, the two groups (Groups A and B) having an increased RC slope showed a significantly higher energy expenditure and power activity in the alpha frequency band at the prefrontal-located EEG electrodes compared to the two groups (Groups C and D) having a decreased RC slope. Possible explanations for these findings will be discussed in the following part.

### Short-Time Synaptic Plasticity – Paired Associative Stimulation

Over all the participants, the RC showed significant increased MEP peak-to-peak amplitudes after PAS (>30 min) visible in the highest stimulation intensity (140% RMT). These enlarged MEP amplitudes indicate an increase in the neuronal excitability induced by increased activation of populations of either cortico-cortical, corticospinal, or spinal motor neurons (or summation of all or some of these). PAS induced short-term neuronal adaptations in form of short-term neuronal plasticity. A reversible increase in the excitability of cortical structures is achieved when a sub-threshold stimulus arrives at the post-synaptic cell before a second supra-threshold stimulus (Mrachacz-Kersting et al., 2007) (Hebb’s law of coincident summation 1949). TMS can be used, in combination with PNS, to experimentally induce short-term synaptic plasticity by PAS, also called Hebbian plasticity (Cirillo et al., 2009). The PAS-based plastic changes of the synaptic structures shows properties that seem to be similar to LTP (Bi and Poo, 1998) as rapid onset, associativity, duration, specificity, and NMDA-receptor dependence (Cooke and Bliss, 2006; Ziemann et al., 2008). PAS as well as motor learning share common neuronal mechanism and, therefore, PAS tests functionally relevant neuronal circuits (Ziemann et al., 2004; Stefan et al., 2006; Rosenkranz et al., 2007; Jung and Ziemann, 2009). Although PAS is one of the most used method, different strategies can be used to induce cortico-cortical and/or corticospinal plasticity, for example, repetitive-TMS and paired-pulse TMS can be also adopted for similar purposes, and, probably, different individual responses can be obtained (Rossini et al., 2007). To sum up, the stimulation of the motor cortex in our older participants and its post-synaptic environment seem to still adapt up to a certain amount to external stimuli. This plastic adaptation is in line with previous studies that showed the possibility of cortical plasticity in older adults triggered by different kind of interventions, e.g., physical and cognitive exercise (Bamidis et al., 2014).

As an increased MEP peak-to-peak amplitude, also an increased RC slope can be used to indicate PAS-induced LTP-like plasticity (Meunier et al., 2007; Rosenkranz et al., 2007). The data-driven automatic hierarchical classification of the individual RC slope led to four different response groups following PAS. Interindividual variability might explain the formation of different response groups after PAS. Several studies focused on interindividual variability following PAS and discussed possible reasons why participants showed different reaction patterns. In our study, the four response groups differ mainly for two factors: RC slope and response duration. For the RC slope, one group (Group A) showed a significant increase after 30 min, which may well indicate PAS-induced LTP-like plasticity (Meunier et al., 2007; Rosenkranz et al., 2007). While another group (Group D) showed a significant decrease of the RC slope after 30 min, which may well indicate PAS-induced LTD-like adaptations. LTD can be generated when the sub-threshold stimulus arrives at the post-synaptic cell after a second supra-threshold stimulus (Hebb’s law of coincident summation 1949) (Ziemann et al., 2008). It might be that the ISI time was too short for those participants as a shorter ISI time can be accompanied by decreased MEP amplitudes (Mrachacz-Kersting et al., 2007). Different heights of the participants and possibly different conduction times for the peripheral nerve and top–down volley are factors that might have contributed to this circumstance (Mrachacz-Kersting et al., 2007) in our older adults. However, no significant height differences were present in this study. Furthermore, the two other groups showed indications for LTP-like (Group B) or LDP-like (Group C) plasticity though the effects appeared already after 5 min and receded 30 min after PAS. Explanations for the short response duration might be that the stimuli were not strong enough to generate a long-lasting effect or the post-synaptic properties of the stimulated motor cortical area were restricted in reacting with minimal plastic adaptations as the magnitude of PAS-induced LTP-like plasticity in human primary motor cortex declines with age (Müller-Dahlhaus et al., 2008; Tecchio et al., 2008). However, no significant age differences were present in this study. In addition to the aforementioned factors, several other factors might be important for interindividual variability including attention stage, time of the day, neuronal motor cortical activity before or during PAS, and endophenotype characteristics (Ziemann et al., 2008). Furthermore, most sources describing PAS approaches have used paradigms in which upper extremities muscles were stimulated as opposed to our paradigm using the TA. Besides, studies showed that lifestyle-based characteristics, e.g., physical activity, influence PAS-induced LTP-like plasticity in the motor cortex (Cirillo et al., 2009; Kumpulainen et al., 2015).

### Relation of Physical Activity, Nutrition, Cognition, and Neurophysiological Functioning to PAS-Induced Plasticity

The comparisons of the assessments including physical activity, nutritional status, cognition, and neurophysiological activity between the four response groups revealed no significant differences. General reasons as well as specific reasons might explain why the assessed factors were not related to the appearance of four different response groups. A reason might be that the used measurement methods were not sensitive enough or their acquisition spectrum was limited to measure their influence on neuronal excitability. For physical activity assessments, the PAQ 50+ is a questionnaire that includes subjective responses that might differ from the real activity behavior. However, the objective assessment using the StepWatch also did not lead to any differences. Motor test batteries (e.g., testing strength or balance) or fitness tests might get a broader scope as several aspects of motor functioning are tested. A previous study showed a differential motor cortical plasticity in skilled and endurance trained athletes that might be because of different training-induced adaptations (Kumpulainen et al., 2015). Another study demonstrated that an association exists between motor skill learning and PAS-induced LTP-like plasticity (Frantseva et al., 2008). For the nutritional assessment, the rationale behind this was that a normal nutritional status should be present to ensure brain plasticity. However, with the exceptions of three participants (21, 21, and 22.5 points; 17 to 23.5 points to being at risk of malnutrition), the participants showed a normal nutritional status. In addition, we have to keep in mind that the questionnaire is not an objective assessment of the nutritional status. The recording of the nutritional status using the questionnaire might be too superficial. Future studies could consider a detailed recording of nutritional intake over a certain period (e.g., by using a diet weekly diary) or blood samples to determine the status of nutrients that are important for the brain, e.g., omega-3 fatty acids. For the cognitive assessment, the direct relation to the primary motor cortex plasticity might be minor to act as a distinctive feature, given that cognitive tests, such as the divided attention task, are well-known to involve mostly the PFC, which is related to executive functioning (Yuan and Raz, 2014). Another reason why no differences for the assessments were observed could be that the grouping was too strict leading to a smaller number of participants in each group and this, in turn, might have limited the detection of any differences. Finally, previous reports indicated that variability in responses to TMS-based neuroplasticity interventions are not uncommon (Fratello et al., 2006; Müller-Dahlhaus et al., 2008; Sale et al., 2008; Japyassu and Malange, 2014; López-Alonso et al., 2014) and controlling all possible contributing factors to this variability is not straightforward (see Ridding and Ziemann, 2010, for an overview). For example, sleep behavior in older adults can be mentioned as a potentially important factor. Maintenance of synapse homeostasis is influenced by slow waves during sleep (Tononi and Cirelli, 2006), which is known to be affected in many older adults (Varga et al., 2016). This may have altered the potentiation capacity of synapses in response to the neuroplasticity intervention (Dickins et al., 2017). Investigation of and trying to control additional factors that influence responses to PAS in older adults might help in reducing the variability in plasticity responses in future studies.

Taking the groups together according to the criteria of RC slope increase or decrease, the two groups (Groups A and B) having a RC slope increase showed a significantly higher energy expenditure than the groups having a decreased RC slope (Groups C and D). The participants with a higher energy expenditure showed an increase of the RC slope that in turn indicates PAS-induced LTP-like plasticity (Meunier et al., 2007; Rosenkranz et al., 2007). However, it has to be remarked that one of the four sub-groups of participants, obtained after the data-driven automatic hierarchical classification, just showed an increase of the RC slope at 5 min after PAS compared to baseline, which receded at 30 min after PAS compared to the first increase. For PAQ 50+ questionnaire, a higher energy expenditure goes hand in hand with a more active lifestyle. Furthermore, the PAQ 50+ might have captured more accurately the physical activity level, within a broader range of lifestyle tasks and exercises of the elderly participants, including housework, gardening, leisure time, sports, and work. The findings of our study seem to be in line with a previous study that demonstrated enhanced corticospinal excitability/plasticity in physically active individuals (determined by a questionnaire) measured for a small hand muscle in young subjects (Cirillo et al., 2009). Based on the results, the researchers suggested that physical activity is an additional factor that can contribute to increased neuroplasticity after PAS and they concluded that regular physical exercise may offer benefits to primary motor cortex functions. Physical exercise can provide a supportive neural environment by increasing the blood flow to the primary motor cortex and this, in turn, is accompanied by increased neurotrophic factors (Klintsova et al., 2004; Vaynman and Gomez-Pinilla, 2005; Adkins et al., 2006). For the questionnaire, however, we should still consider that the energy expenditure was calculated in terms of subjective responses of the participants and we recommend a more objective assessment of energy expenditure and physical activity for future studies.

Furthermore, the older adults with a RC slope increase (Groups A and B) showed significant higher EEG alpha power at Fp1 channel and a (non-significant) upward trend at the contralateral channel compared to the two groups (Groups C and D) having a RC slope decrease. Physiological aging is usually linked to a general decrease in EEG power within the alpha frequency band, which is most pronounced on the occipital area. Nevertheless, in aging the distribution of EEG alpha power seems to shift toward most frontal area as well (Niedermeyer, 1997; Babiloni et al., 2006; Rossini et al., 2007). Interestingly, next to alterations in cortical oscillatory activity, studies showed that getting older can be accompanied by physiological cortical atrophy, especially for the frontal lobe (Raz et al., 2005), and this, in turn, might also affect neuronal excitability because of the increased distance to the excitable elements (Rossini et al., 2007). As mentioned before, the older adults who were more physically active showed an increase of the RC slope. Thus, it might be that both neuronal excitability and activity benefit from the physically active lifestyle by possibly counteracting the physiological aging processes occurring at cortical level. Regarding brain aging, physical activity can strengthen neuronal structures, synaptic plasticity, and neuronal transmission (Cai et al., 2014) and, thus, being important for triggering molecular and cellular mechanisms in order to counteract age- and behavior-related neuroanatomical and neurophysiological decline (Cotman and Berchtold, 2002; Voss et al., 2013; Phillips et al., 2015). EEG power in the alpha frequency band increased during the auditory stimuli of the divided attention task. This might be due to the fact that perceptual operations of acoustic inputs are encoded by working memory and these processes are affected by age-limited processing resources (Wingfield et al., 2005). The older adults of our study might still have good working memory abilities so that sufficient processing resources are available for acoustic inputs. A study showed that frontal alpha peak frequency was positively associated with working memory performance independent of age (Richard Clark et al., 2004). In addition, a larger enhancement of frontal alpha in response to auditory stimuli in older adults has been reported (Yordanova et al., 1998). To sum up, a connection might exist between a physically active lifestyle, the capacity of generating LTP-like synaptic plasticity, and the levels of EEG power in the alpha frequency band in healthy older adults. Future studies might examine the behavior of EEG power in similar settings involving nutrition, cognition, and corticospinal excitability/plasticity, but in older adults showing either a sedentary or a physically active lifestyle or after a training intervention involving only motor, cognitive, or combined motor-cognitive components.

### Limitations

Several factors might have limited this study. The measurements were just a snapshot of the participants’ characteristics/life style factors and their corticospinal functioning. Consideration of specific predispositions not taken into account by us (e.g., previous physical training, educational status, etc.), a longer observation period and qualitative assessment of both physical activity and nutrition might have provided a more accurate specification regarding to physical activity, nutritional status, and cognition. Another limitation might be that the TMS measurements of the individual participants were not performed at the same time of day (Ziemann et al., 2008). Furthermore, it cannot be excluded that the measured adaptations were generated on cortical level, as PAS protocols may well-induce changes on either supra-spinal and sub-cortical level (or both), although, the used ISI was determined by a previous study to be long enough to reach the cortical structures (Kumpulainen et al., 2012). Individual ISI time determination is possible by measuring the somatosensory-evoked potential and the central processing time resulting in P32 + 18 ms for the leg area (Kumpulainen et al., 2012). Thus, future studies should consider the inter-individual variability that can affect the response to the PAS protocol. Moreover, also different strategies to induce plasticity at cortical level should be considered (i.e., repetitive TMS, paired-pulse TMS). On the neurophysiological assessment, it should be noted that in this study a low-density EEG device was used. Future studies should consider employing a high-density EEG device (i.e., 64 or more channels), which can allow a broader coverage of the cortical activity as well as additional analyses on source space.

## Conclusion

This study is one of the first that considered exploration of several factors that may be related to PAS-induced plasticity of the motor cortex in older adults. The results of this study indicated that older adults differ in the capacity to generate short-time synaptic plasticity using the PAS protocol. Some participants were able to generate short-time synaptic plasticity as demonstrated by increased MEP peak-to-peak amplitudes and steeper RC slopes. A connection might exist between a physically active lifestyle, EEG power in the alpha frequency band over the PFC and the capacity of generating LTP-like plasticity in the motor cortex in healthy older adults. A plastic brain and a supportive neuronal environment, especially for the primary motor cortex, is important for proper motor functioning. Intact motor functioning is crucial to preserve an active lifestyle in older adults and, to close the circle, physical activity influences molecular and cellular mechanisms counteracting age- and behavior-related neuroanatomical and neurophysiological decline (Cotman and Berchtold, 2002; Voss et al., 2013; Phillips et al., 2015). Future studies should consider different assessments and approaches that might be more sensitive. Moreover, future studies should consider bigger sample sizes to get a broader scope of the older adults’ population to identify differences according to the capacity of synaptic plasticity.

## Author Contributions

AS, ME, and SV developed the research question under the lead of EDB. The concept and design part was established by AS, ME, and SV while EDB acted as methodological council. AS, ME, and SV did data acquisition, analysis and interpretation of the results, which was edited and improved by EDB. FG performed the EEG data analysis and interpretation of the results. AS, FG, and EDB substantially revised the manuscript to bring it to its current version. All authors have read and approved the final manuscript.

## Conflict of Interest Statement

The authors declare that the research was conducted in the absence of any commercial or financial relationships that could be construed as a potential conflict of interest.

## Abbreviations

BDNF: brain-derived neurotrophic factor
CMS: common mode sense
DRL: driven right leg
EEG: electroencephalography
EF: executive function
EHI: Edinburgh Handedness Inventory
EMG: electromyography
GDS: Geriatric Depression Scale
ISI: interstimulus interval
LTD: long-term depression
LTP: long-term potentiation
MEP: motor-evoked potential
MET: metabolic equivalents of task
MNA: Mini Nutritional Assessment
MoCA: Montreal Cognitive Assessment
NMDA: *N*-methyl-D-aspartate
PAQ: Physical Activity Questionnaire
PAS: paired associative stimulation
PFC: prefrontal cortex
PNS: peripheral nerve stimulation
RC: recruitment curve
RMT: resting motor threshold
SD: standard deviation
TA: tibialis anterior
TAP: Test of Attentional Performance
TMS: transcranial magnetic stimulation.
