# Supplementary material for: Physical Activity, Nutrition, Cognition, Neurophysiology, and Short-Time Synaptic Plasticity in Healthy Older Adults: A Cross-Sectional Study
Source: Front Aging Neurosci. 2018 Aug 30;10:242. doi: 10.3389/fnagi.2018.00242 (PMC6125692; doi:10.3389/fnagi.2018.00242)
Supplement: Supplementary file 1 [file Table_1.docx]

Physical Activity, Nutrition, Cognition, Neurophysiology and Short-Time Synaptic Plasticity in Healthy Older Adults: A Cross-Sectional Study

Alexandra Schättin^1*^, Federico Gennaro^1^, Martin Egloff^1^, Simon Vogt^1^, Eling D. de Bruin^1,2^

^1^Department of Health Sciences and Technology, Institute of Human Movement Sciences and Sport, ETH Zürich, HCP, Leopold-Ruzicka-Weg 4, 8093 Zurich, Switzerland

^2^ Department of Neurobiology, Care Sciences and Society, Karolinska Institutet, Alfred Nobels Alle 23, 141 83 Huddinge, Sweden

# Supplementary table 1

**Table 1. Comparison of normalized motor evoked potential peak-to-peak amplitudes for before, 5min after, and 30min after paired associative stimulation.**

|  | **Before** | **5min after** | **30min after** | χ^2^ | df | p |
| --- | --- | --- | --- | --- | --- | --- |
| **90% RMT** | 0.08 (0.03; 0.19) | 0.07 (0.03; 0.18) | 0.11 (0.03; 0.18) | 0.222 | 2 | 0.895 |
| **100% RMT^1^** | 0.20 (0.10; 0.29) | 0.19 (0.12; 0.36) | 0.21 (0.13; 0.50) | 4.800 | 2 | 0.091 |
| **110% RMT** | 0.36 (0.21; 0.67) | 0.46 (0.22; 0.68) | 0.35 (0.22; 0.73) | 2.800 | 2 | 0.247 |
| **120% RMT** | 0.62 (0.29; 0.80) | 0.58 (0.39; 0.81) | 0.72 (0.46; 0.86) | 3.389 | 2 | 0.184 |
| **130% RMT** | 0.84 (0.71; 0.99) | 0.87 (0.65; 1.06) | 0.97 (0.65; 1.06) | 6.000 | 2 | 0.050* |
| **Post-hoc** | **z** | **p_adapted_** | **r** |  |  |  |
| Before – 5min after | -2.121 | 0.102 | 0.25 |  |  |  |
| 5min after – 30min after | < 0.001 | 0.999 | < 0.01 |  |  |  |
| Before – 30min after | -2.121 | 0.102 | 0.25 |  |  |  |
| **140% RMT** | 1.00 (1.00; 1.00) | 0.95 (0.80; 1.19) | 1.09 (1.02; 1.22) | 13.556 | 2 | 0.001* |
| **Post-hoc** | **z** | **p_adapted_** | **r** |  |  |  |
| Before – 5min after | 0.236 | 0.999 | 0.03 |  |  |  |
| 5min after – 30min after | -3.300 | 0.003* | 0.39 |  |  |  |
| Before – 30min after | -3.064 | 0.007* | 0.36 |  |  |  |

N = 36, ^1^N = 35. Motor evoked potential values were normalized to the individual maximum (140%) pre-measurement RMT. Normalized data are median values (interquartile range) as indicated. For each stimulation intensity, the comparison of before, 5min after, and 30min after PAS was performed using Friedman test. Dunn-Bonferroni post-hoc test was performed when a significant comparison resulted. *p ≤ 0.05. For effect size, r = 0.1 is considered a “small” effect, around 0.3 a “medium” effect, and 0.5 and above a “large” effect. RMT = resting motor threshold.
